# Supplementary material for: Biferroelectricity of a homochiral organic molecule in both solid crystal and liquid crystal phases
Source: Nat Commun. 2022 Oct 18;13:6150. doi: 10.1038/s41467-022-33925-2 (PMC9579164; doi:10.1038/s41467-022-33925-2)
Supplement: Supplementary file 1 — Supplementary Information [file 41467_2022_33925_MOESM1_ESM.pdf]

Supplementary Information

Biferroelectricity of a homochiral organic molecule in both solid crystal and liquid crystal phases

Xian-Jiang Song,[1,2] Xiao-Gang Chen,[1,2] Jun-Chao Liu,[1,2] Qin Liu,[1] Yi-Piao Zeng,[1] Yuan-Yuan Tang,[1] Peng-Fei Li,[1] Ren-Gen Xiong[1]* and Wei-Qiang Liao[1]*

[1]Ordered Matter Science Research Center, Nanchang University, Nanchang 330031, People's Republic of China.

[2]These authors contributed equally: Xian-Jiang Song, Xiao-Gang Chen, Jun-Chao Liu.

*e-mail: xiongrg@seu.edu.cn; liaowq@ncu.edu.cn

Measurement Methods

Synthesis and preparation of Materials.

Synthesis of 4-iodocinnamic acid.

4-Iodocinnamic acid was synthesized according to the literature procedures.[1] Malonic acid (3.12 g, 30.0 mmol) and piperidine 6 mmol) were added to a solution of 4-iodobenzaldehyde (4.64 g, 20.0 mmol) in pyridine (80 mL). The reaction mixture was refluxed for 4 h. After the reaction was completed, the resultant mixture was acidified with 2 mol/L HCl to adjust the pH to 1~2 and then extracted with ethyl acetate (100 mL × 5). The organic layer was washed with deionized water, fully dried over with anhydrous $MgSO_4$, concentrated, and dried in a vacuum to give a white solid. 4-Iodocinnamic acid obtained above was used directly in the following reaction without further purification. The synthesis route of 4-iodocinnamic acid is shown in Supplementary Fig. 22.

Synthesis of *β*-sitosteryl 4-iodocinnamate (4I-CASS).

β-Sitosterol (2,76 g, 5 mmol, 75%), 4-iodocinnamic acid (1.37 g, 5 mmol), DMAP (2.20 g, 18 mmol), and DCC (3.09 g, 15 mmol) were added to a closed pressure-resistant bottle with 45 mL DCM, and

the mixture was stirred at 90 °C. After 48 hours, the resultant mixture was returned to room temperature, filtered, washed with DCM, and the solvent was removed under reduced pressure. The crude product was then purified by column chromatography (DCM / petroleum ether (PE) = 1: 5) to obtain a white solid. The synthesis route of 4I-CASS is shown in Supplementary Fig. 23.

Synthesis of cholesteryl 4-iodocinnamate (4I-CACS).

Cholesterol (1.93 g, 5 mmol), 4-iodocinnamic acid (1.37g, 5 mmol), 4-dimethylaminopyridine (DMAP, 2.20 g, 18 mmol), dicyclohexylcarbodiimide (DCC, 3.09 g, 15 mmol) were added to a closed pressure-resistant bottle filled 45 mL dichloromethane (DCM), and the mixture was stirred at 90 °C for 48 hours. After the completion of the reaction, the resultant mixture was filtered, washed with DCM, and the solvent was removed under reduced pressure. The crude product was then purified by column chromatography (DCM / PE = 1:5) to obtain a white solid. The synthesis route of 4I-CACS is shown in Supplementary Fig. 24.

Synthesis of dihydrocholesteryl 4-iodocinnamate (4I-CAHCS).

Dihydrocholesterol (1.94 g, 5 mmol), 4-iodocinnamic acid (1.37 g, 5 mmol), DMAP (2.20 g, 18 mmol), and DCC (3.09 g, 15 mmol) were added to a closed pressure-resistant bottle with 45 mL DCM, and the mixture was stirred at 90 °C for 48 hours. Then, the resultant mixture was cooled, filtered, washed with DCM, and the solvent was removed under reduced pressure. The crude product was then purified by column chromatography (DCM / PE = 1: 5) to obtain a white solid. The synthesis route of 4I-CAHCS is shown in Supplementary Fig. 25.

Single-crystal X-ray crystallography. Single-crystal X-ray diffraction data of 4I-CASS, 4I-CACS, and 4I-CAHCS were measured using a Rigaku Saturn 924 diffractometer with Mo-Kα radiation (λ = 0.71073 Å). Data collection, cell refinement, and data reduction was performed using Rigaku CrystalClear 1.3.5. The structures were solved by direct methods and refined by the full-matrix method based on F^2 using the Olex2 and SHELXTL software package. All non-hydrogen atoms were refined anisotropically and the positions of all hydrogen atoms were generated geometrically. The data collection and structure refinement of these crystals are summarized in Supplementary Table 1. CCDC numbers: 2189236-2189239.

Powder X-ray diffraction. Powder X-ray diffraction (PXRD) data were measured using a Rigaku D/MAX 2000 PC X-ray diffraction system with Cu Kα radiation in the 2θ range of 5°–50° with a step size of 0.02°.

DSC and dielectric measurements. Differential scanning calorimetry (DSC) measurements were recorded on a NETZSCH DSC 200F3 instrument by heating and cooling crystalline samples with a rate of 10 K min^{-1} under aluminum crucibles at nitrogen atmosphere. Complex dielectric permittivities were measured with a TH2828A impedance analyzer. Silver conductive paste deposited on the plate surfaces of samples were used as top and bottom electrodes.

Calculation condition. The geometry optimization, vibration analysis and dipole moment were calculated at b3lyp/6-31G(d) level with Gaussian 16 software. DFT-D dispersion correction was treated with DFT-D3 method with Becke-Jonson damping[2]. We constructed molecular conformation based on the experimentally measured single crystal X-ray diffraction structure. We carried out density functional calculations based on the Berry phase method developed by Kingsmith and Vanderbilt.[3,4] The first-principles calculations were performed within the framework of density functional theory implemented in the Vienna ab initio Simulation Package (VASP; 5.4.4).[5,6] The energy cut-off for the expansion of the wave functions was fixed to 550 eV and the exchange−correlation interactions were treated within the generalized gradient approximation of the Perdew−Burke−Ernzerhof type.[7] Van der Waals corrections are calculated based on DFT-D3 method with Becke-Jonson damping. Firstly, the geometrical optimization was performed by fixing the lattice constant based on the experimentally X-ray crystal structure until the change of the total energy is smaller than 0.001. Then, the Berry phase calculation was employed based on the optimized geometry.

Polarization-voltage loop measurements.

SmC* phase. Commercial liquid cell with polyimide alignment layer was used in the test with area of 1 cm^2 and thickness of 4 μm. 4I-CASS was filled into the liquid cell above clearing point by capillary action. For the measurement of polarization reversal current, triangle wave method (TWM) was performed on a Precision Multiferroic II instrument (Radiant Technologies Inc.). The applied voltage and period of triangle wave were 100 V and 90 ms, respectively, and the temperature under test was 155 °C in SmC* phase. The current curve was composed of ohmic, capacitive and polarization reversal

contributions. The first two contributions were separated from the curve and integration of the positive and negative reversal peaks yielded the polarization-voltage loop.

Phase I. Sample (5 mg) was first dissolved in ethyl acetate (1 mL), then 20 μL of the solution was dropped on the glass substrate coated with ITO to form the film. Double wave method (DWM) was utilized to determine the polarization reversal current by a voltage source (Trek 609E-6), waveform generator (Keysight 33500B) and current meter (Keithley 6517B). During the test, a needle was contacted with ITO coating and another needle dipped with liquid InGa alloy was placed on the sample film to form a circuit. The polarization value was obtained by integrating the reversal current peak and dividing the area of liquid InGa alloy droplet.

PFM characterization. The PFM measurements were carried out on a commercial piezoresponse force microscope (Oxford instrument, Cypher ES) with high-voltage package and in-situ heating stage. PFM is based on the atomic force microscopy (AFM), with an AC drive voltage applied to the conductive tip. Conductive Pt/Ir-coated silicon probes (EFM, Nanoworld) were used for domain imaging and polarization switching studies, with a nominal spring constant of ~2.8 nN/nm and a free-air resonance frequency of ~75 kHz. Since the amplitude of the low-frequency vertical PFM was within the noise level of the quadrant photodetector of the AFM, we performed the PFM experiments at contact resonance.

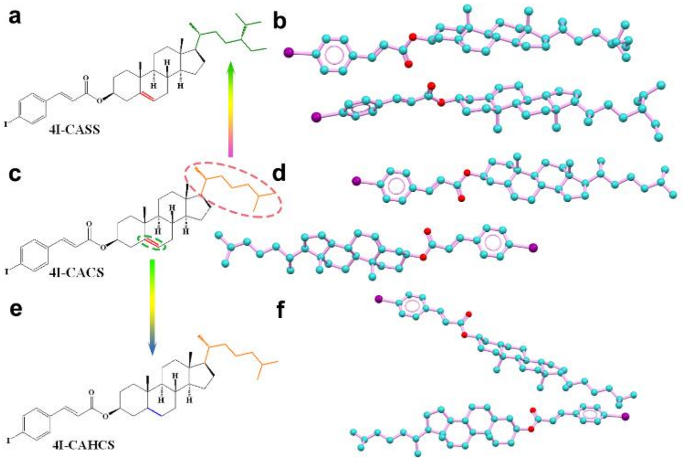

Supplementary Fig. 1 | Comparison of the molecular structure and the asymmetric unit of the crystal structure. The molecular structure and the asymmetric unit of the crystal structure of 4I-CASS (a and b), 4I-CACS (c and d), and 4I-CAHCS (e and f) at 300 K.

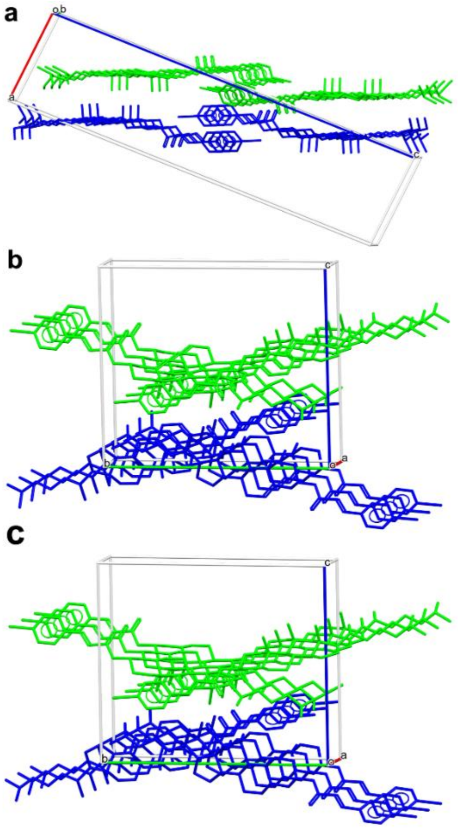

Supplementary Fig. 2 | Comparison of the packing view of crystal structures of 4I-CASS, 4I-CACS, and 4I-CAHCS. The packing view of the crystal structure of 4I-CASS (a), 4I-CACS (b), and 4I-CAHCS (c) at 300 K. The two different colors stand for two kinds of crystallographically independent molecules.

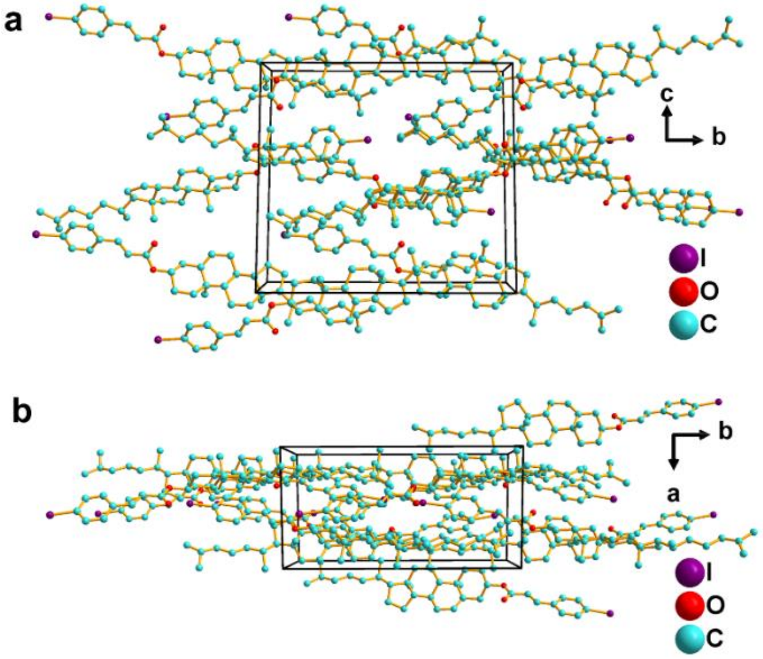

Supplementary Fig. 3 | Packing view of crystal structures of 4I-CACS. (**a**) Packing view along the [1 0 0] direction. (**b**) Packing view along the [0 0 1] direction.

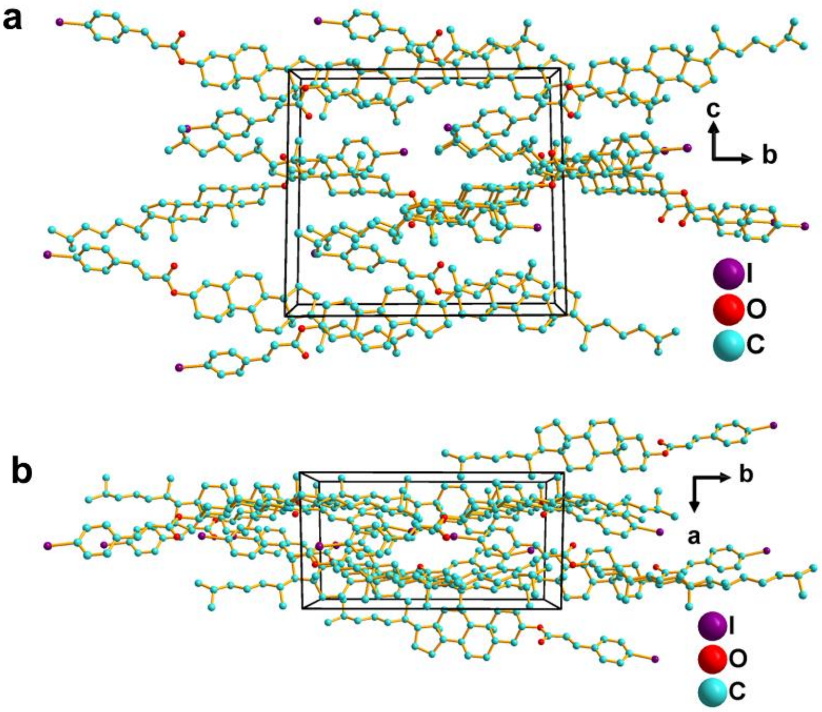

Supplementary Fig. 4 | Packing view of crystal structures of 4I-CAHCS. (**a**) Packing view along the [1 0 0] direction. (**b**) Packing view along the [0 0 1] direction.

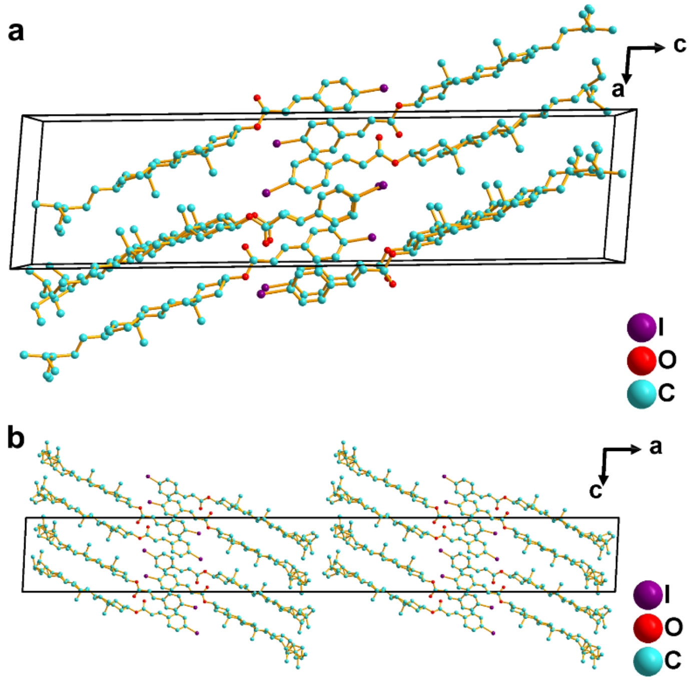

Supplementary Fig. 5 | Packing view of crystal structures of 4I-CASS. (**a**) Packing view in phase I along the [0 1 0] direction. (**b**) Packing view in phase II along the [0 1 0] direction.

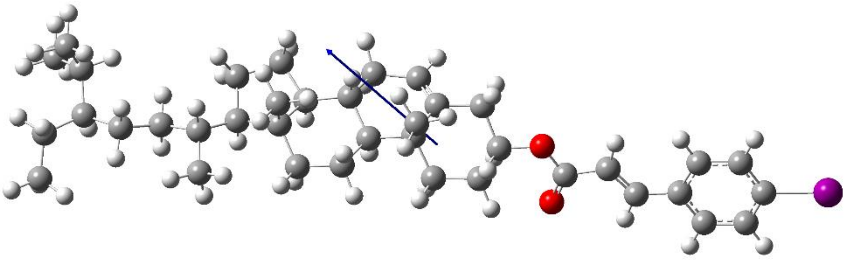

Supplementary Fig. 6 | Molecular dipole of 4I-CASS in phase I. Calculated molecular configuration of 4I-CASS in phase I with 1.82 Debye. The blue arrow indicates the dipole direction.

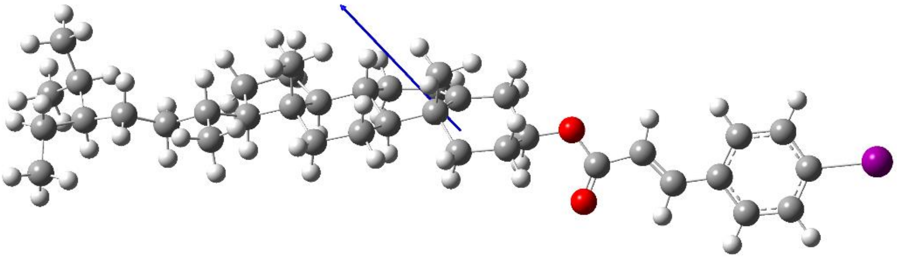

Supplementary Fig. 7 | Molecular dipole of 4I-CASS in phase II. Calculated molecular configuration of 4I-CASS in phase II with 1.82 Debye. The blue arrow indicates the dipole direction.

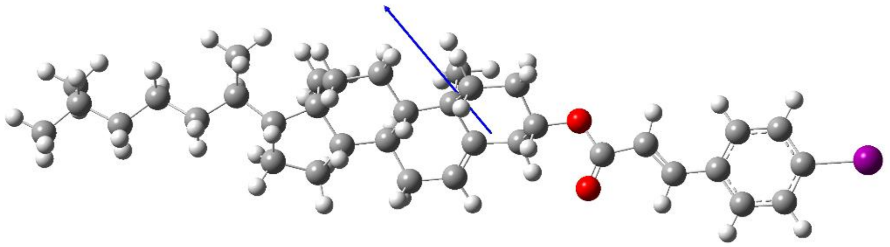

Supplementary Fig. 8 | Molecular dipole of 4I-CACS. Calculated molecular configuration of 4I-CACS with 1.85 Debye. The blue arrow indicates the dipole direction.

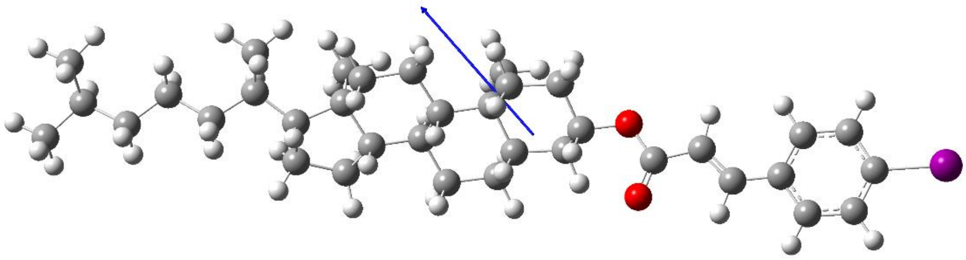

Supplementary Fig. 9 | Molecular dipole of 4I-CAHCS. Calculated molecular configuration of 4I-CAHCS with 1.82 Debye. The blue arrow indicates the dipole direction.

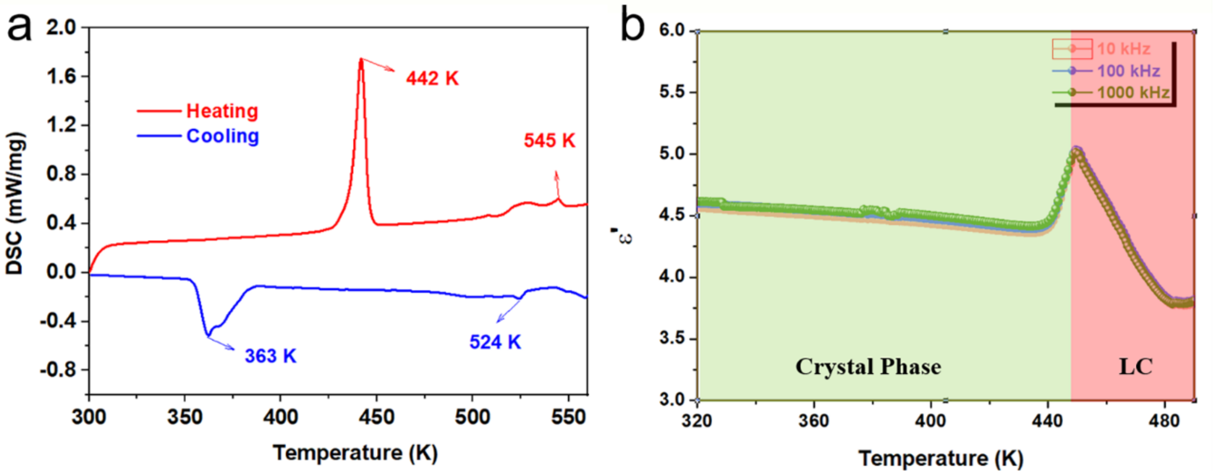

Supplementary Fig. 10 | DSC and dielectric analysis of 4I-CACS. DSC curve (**a**) and temperature-dependent ε' (**b**) of 4I-CACS.

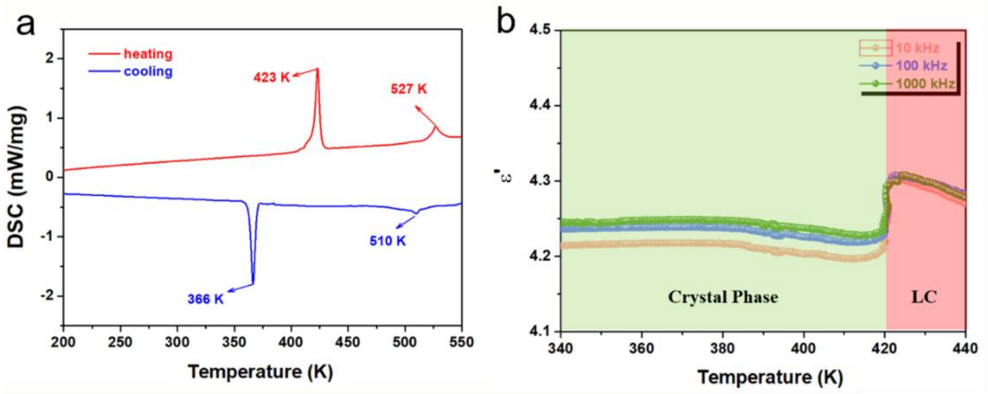

Supplementary Fig. 11 | DSC and dielectric analysis of 4I-CAHCS. DSC curve (**a**) and temperature-dependent *ε′* (**b**) of 4I-CAHCS.

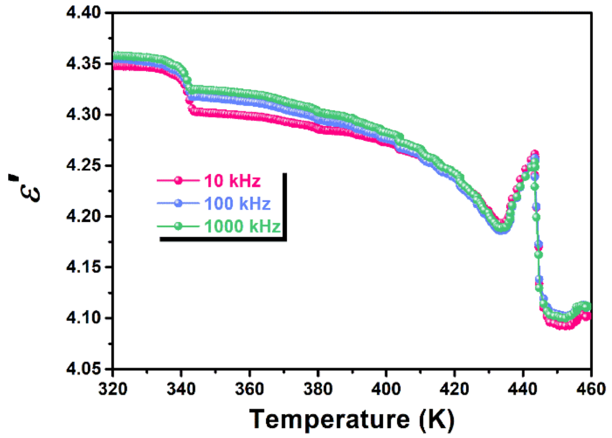

Supplementary Fig. 12 | Temperature-dependent *ε′* of 4I-CASS. The *ε′* of 4I-CASS as a function of temperature at selected frequency.

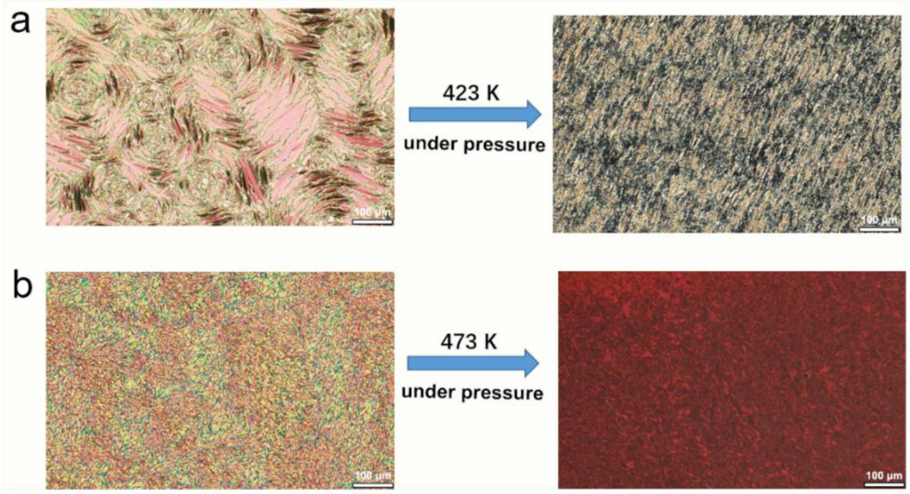

Supplementary Fig. 13 | Polarized photomicrographs of 4I-CASS. Polarized photomicrographs of 4I-CASS under pressure changes at 423 K (**a**) and 473 K (**b**). The focal conic texture of cholesteric phase transforms into oil filament texture under pressure.

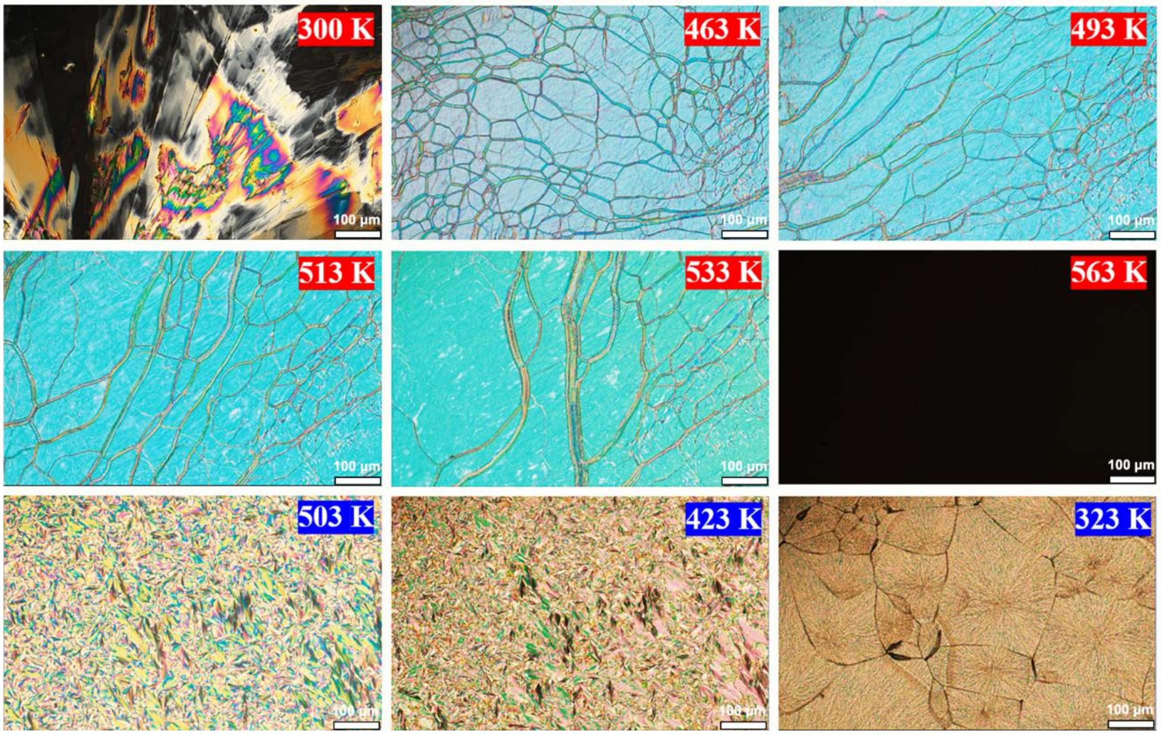

Supplementary Fig. 14 | Polarized photomicrographs of 4I-CACS. Polarized photomicrographs of 4I-CACS in the heating and cooling runs.

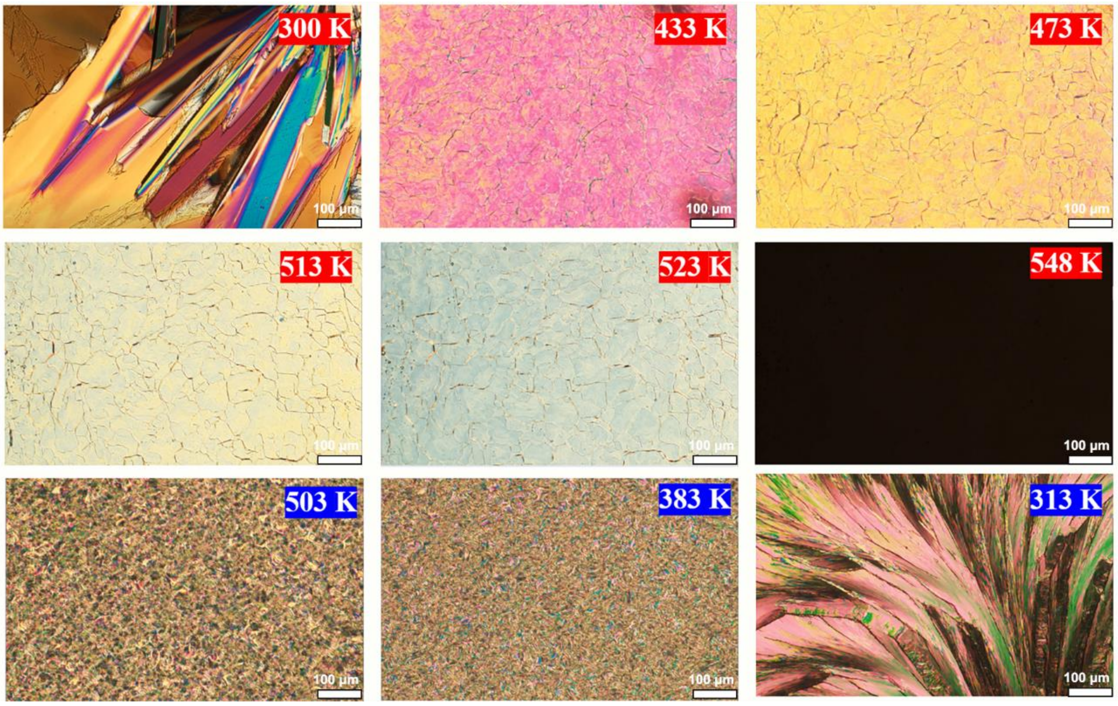

Supplementary Fig. 15 | Polarized photomicrographs of 4I-CAHCS. Polarized photomicrographs of 4I-CAHCS in the heating and cooling runs.

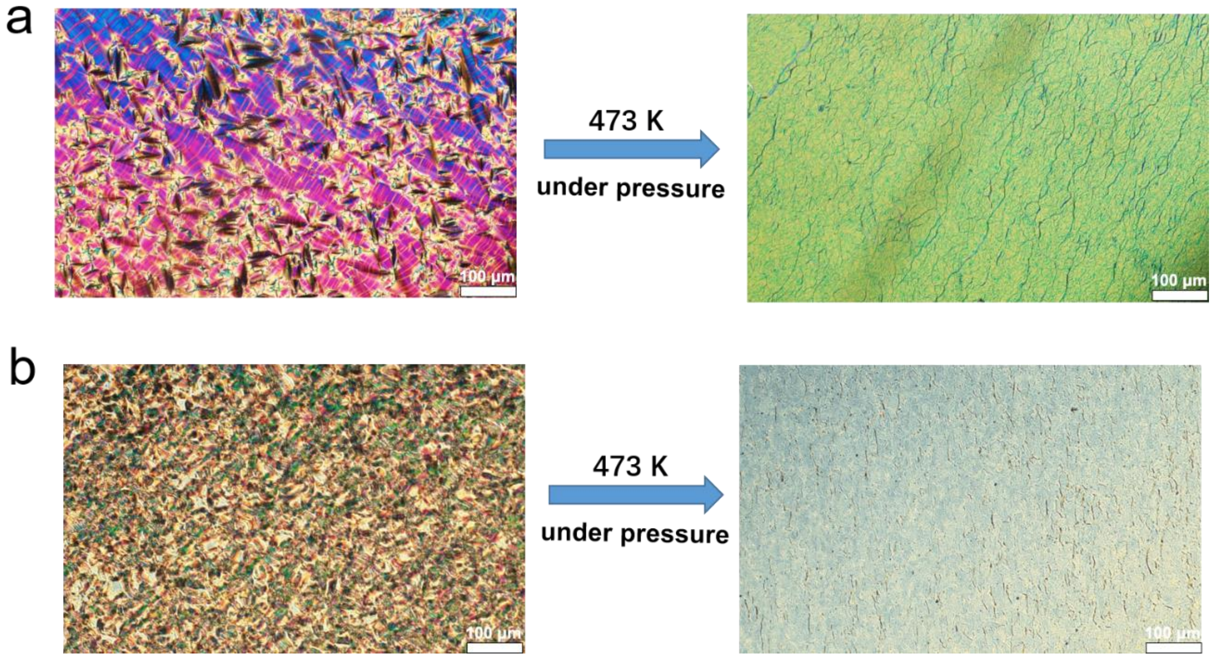

Supplementary Fig. 16 | Polarized photomicrographs of 4I-CACS and 4I-CAHCS. Polarized photomicrographs of 4I-CACS (**a**) and 4I-CAHCS (**b**) under pressure changes.

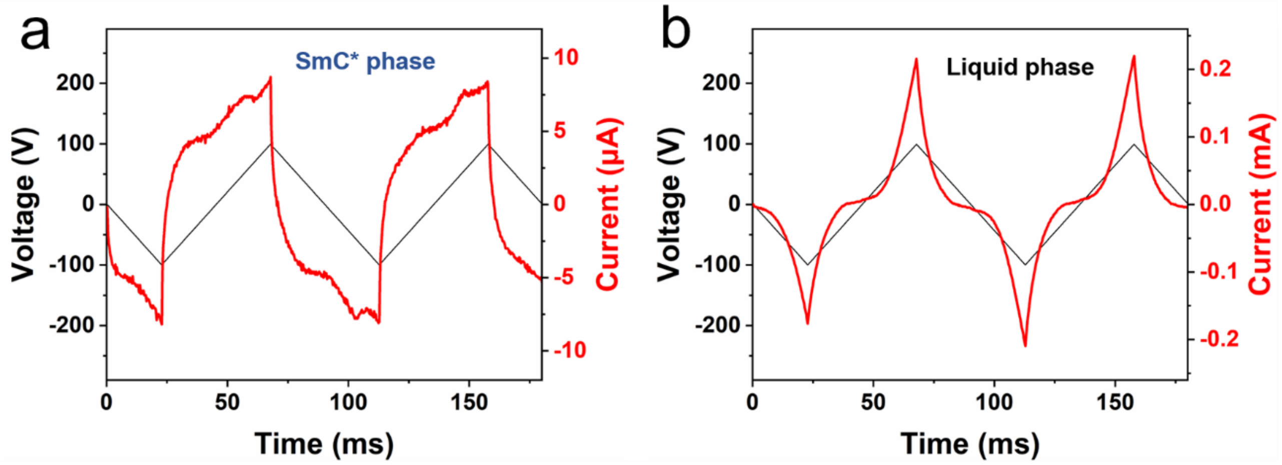

Supplementary Fig. 17 | Current response of 4I-CASS. The measured characteristic current response of 4I-CASS in SmC* phase (**a**) and liquid phase (**b**).

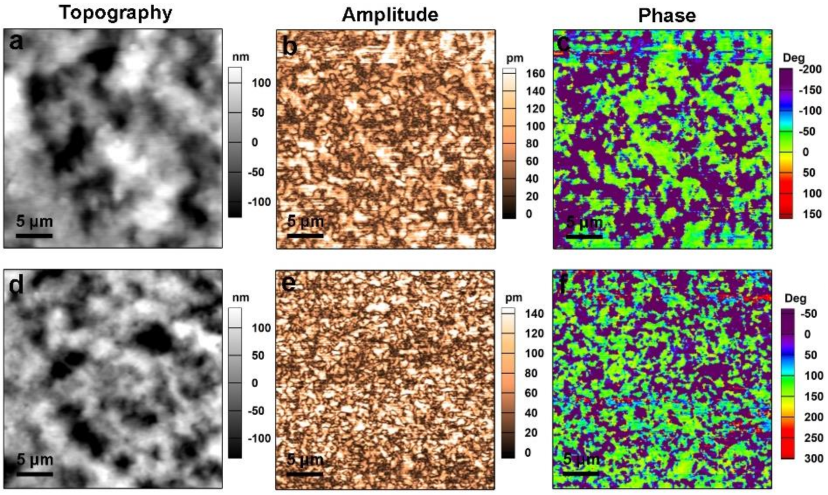

Supplementary Fig. 18 | Domain structures of 4I-CASS. Domain structures observed in different regions of the same film of 4I-CASS.

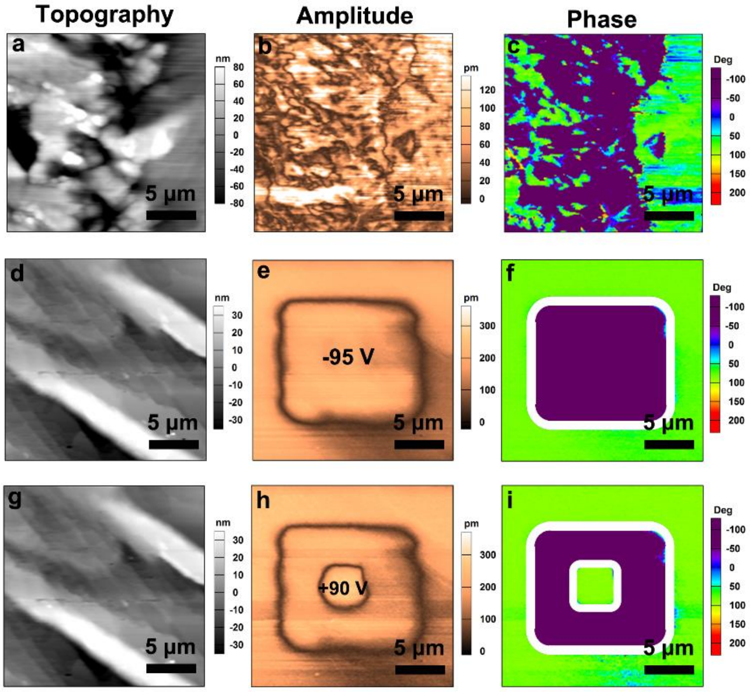

Supplementary Fig. 19 | Domain structures and domain switching of the 4I-CASS thin film in phase II. a-c, Topographic (**a**), lateral PFM amplitude (**b**) and phase (**c**) images mapped on the spin-coating thin film. **d-f**, Topography (**d**), vertical PFM amplitude (**e**) and phase (**f**) images after applying a tip voltage of -95 V in the center area of a region with an initial state of a single domain state. **g-i**, Topography (**g**), vertical PFM amplitude (**h**) and phase (**i**) images after applying +90 V tip voltage in the center region of the switched domain.

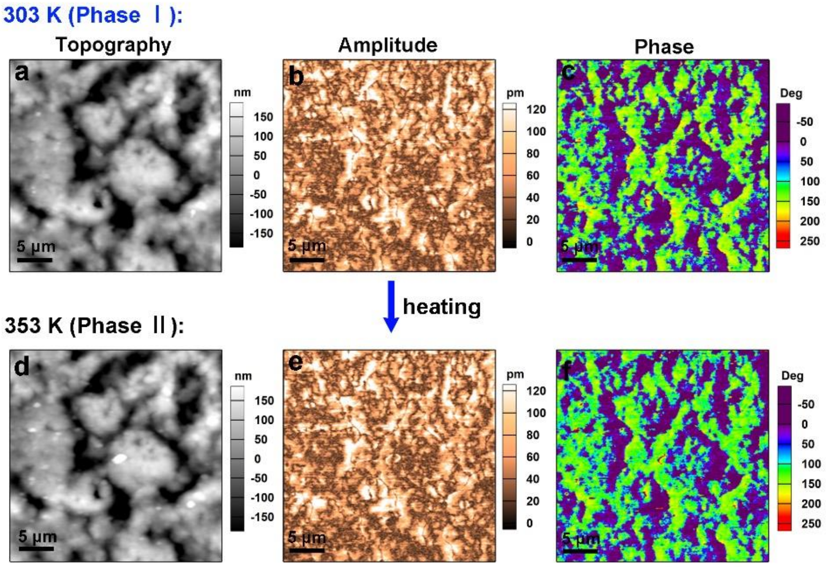

Supplementary Fig. 20 | Domain evolution with temperature of 4I-CASS. The domain patterns did not change significantly from phase I to phase II.

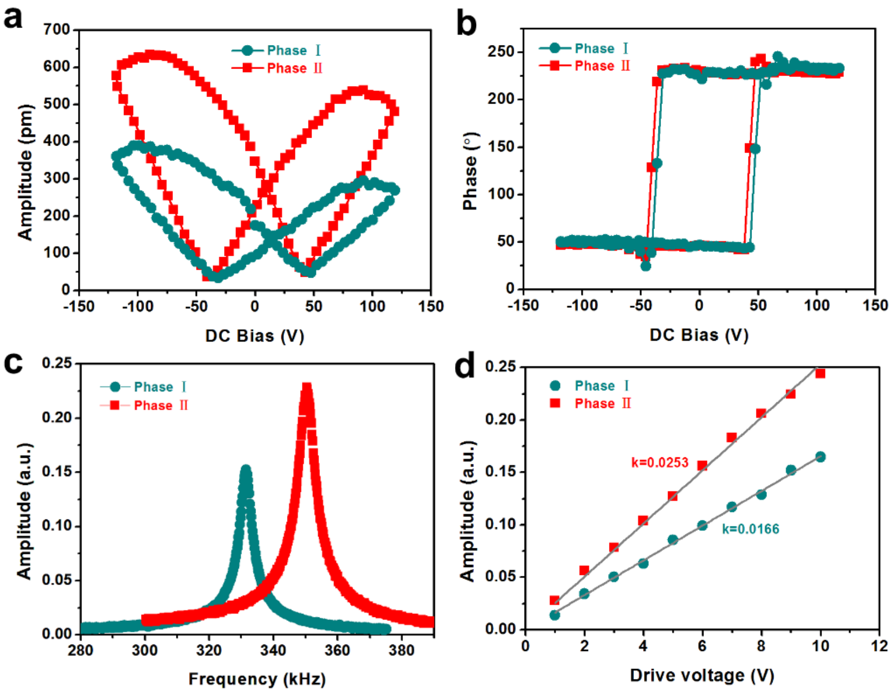

Supplementary Fig. 21 | Piezoelectric response of 4I-CASS thin film in phase I and phase II. DC bias-dependent amplitude (**a**) and phase (**b**) loops of 4I-CASS thin film in phase I and phase II. **c**, Piezoelectric response versus AC frequency curves for the thin film in phase I and phase II. **d**, Peizoelectric amplitude of 4I-CASS thin film was plotted against the excitation bias in phase I and phase II.

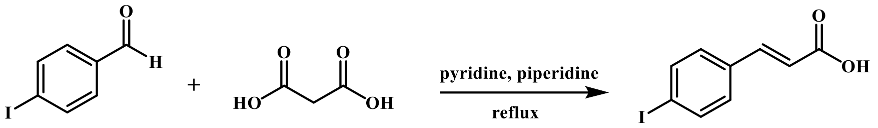

Supplementary Fig. 22 | Synthesis route of 4-iodocinnamic acid. The synthesis route for preparing 4-iodocinnamic acid.

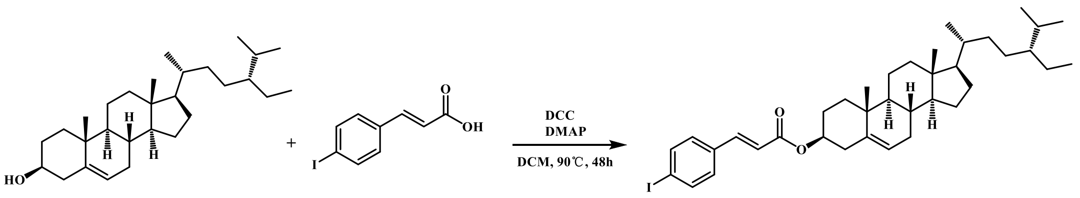

4I-CASS

Supplementary Fig. 23 | Synthesis route of 4I-CASS. The synthesis route for preparing 4I-CASS. The DCC, DMAP, and DCM represent the dicyclohexylcarbodiimide, 4-dimethylaminopyridine, and dichloromethane, respectively.

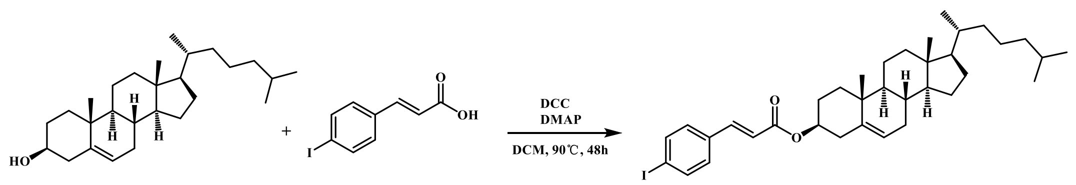

4I-CACS

Supplementary Fig. 24 | Synthesis route of 4I-CACS. The synthesis route for preparing 4I-CACS. The DCC, DMAP, and DCM represent the dicyclohexylcarbodiimide, 4 dimethylaminopyridine, and dichloromethane, respectively.

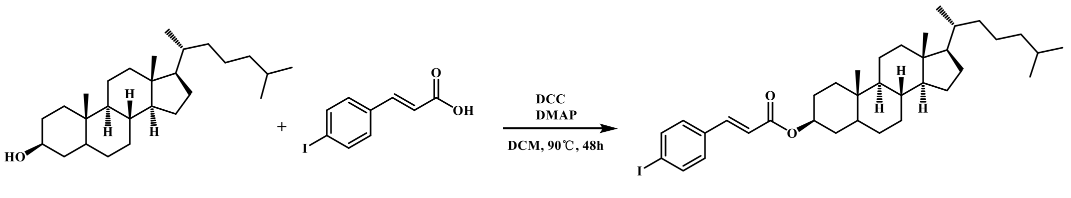

4I-CAHCS

Supplementary Fig. 25 | Synthesis route of 4I-CAHCS. The synthesis route for preparing 4I-CAHCS. The DCC, DMAP, and DCM represent the dicyclohexylcarbodiimide, 4-dimethylaminopyridine, and dichloromethane, respectively.

Supplementary Table 1. Crystal data and structure refinement for 4I-CASS at 300 K, 4I-CASS at 363 K, 4I-CACS at 300 K and 4I-CAHCS at 300 K, respectively.

**Compound**	4I-CASS	4I-CASS	4I-CACS	4I-CAHCS
Temperature	300 K	363 K	300 K	300 K
Formula	$2(C_{38}H_{55}IO_2)$	$2(C_{38}H_{55}IO_2)$	$2(C_{36}H_{51}IO_2)$	$2(C_{36}H_{53}IO_2)$
Weight	1341.43	1341.43	1284.32	1289.36
System	Monoclinic	Monoclinic	Monoclinic	Monoclinic
Space group	$P2_1$	$C2$	$P2_1$	$P2_1$
a (Å)	10.7811(4)	87.404(4)	10.0728(2)	10.3482(4)
b (Å)	7.6568(2)	7.6192(5)	19.3074(3)	19.2883(3)
c (Å)	42.9796(11)	10.9472(8)	17.8140(3)	17.6453(5)
β (°)	95.100(3)	92.800(6)	103.419(2)	103.561(3)
V (Å^3)	3533.87(18)	7281.5(8)	3369.87(11)	3423.80(18)
Z	2	4	2	2
R_{int}	0.0531	0.0802	0.0852	0.0153
R_1	0.0732	0.1426	0.0745	0.0811
wR_2	0.2406	0.3784	0.2214	0.2405
GOF	1.087	1.336	1.023	1.067

Supplementary Table 2. Calculated dipole moment for single molecule and ferroelectric polarization for each crystal.

Crystal	4I-CASS at 300 K	4I-CASS at 363 K	4I-CACS at 300 K	4I-CAHCS at 300 K
Dipole moment (Debye)	1.82	1.82	1.85	1.82
Polarization (nC/cm^2)	181.93	233.17	27.12	28.53

Supplementary References

1. Li, F.-N. *et al.* Design, synthesis, and biological evaluation of novel diarylalkyl amides as TRPV1 antagonists. *Biorg. Med. Chem.* **17**, 3557-3567 (2009).
2. Grimme, S., Ehrlich, S. & Goerigk, L. Effect of the Damping Function in Dispersion Corrected Density Functional Theory. *J. Comput. Chem.* **32**, 1456-1465 (2011).
3. King-Smith, R. & Vanderbilt, D. Theory of polarization of crystalline solids. *Phys. Rev. B* **47**, 1651 (1993).
4. Vanderbilt, D. & King-Smith, R. Electric polarization as a bulk quantity and its relation to surface charge. *Phys. Rev. B* **48**, 4442 (1993).
5. Kresse, G. & Furthmüller, J. Efficient iterative schemes for ab initio total-energy calculations using a plane-wave basis set. *Phys. Rev. B* **54**, 11169 (1996).
6. Kresse, G. & Furthmüller, J. Efficiency of ab-initio total energy calculations for metals and semiconductors using a plane-wave basis set. *Comput. Mater. Sci.* **6**, 15-50 (1996).
7. Perdew, J. P., Burke, K. & Ernzerhof, M. Generalized Gradient Approximation Made Simple. *Phys. Rev. Lett.* **77**, 3865-3868 (1996).
